# Disentangling in vivo the effects of iron content and atrophy on the ageing human brain

**DOI:** 10.1016/j.neuroimage.2014.09.044

**Published:** 2014-12

**Authors:** S. Lorio, A. Lutti, F. Kherif, A. Ruef, J. Dukart, R. Chowdhury, R.S. Frackowiak, J. Ashburner, G. Helms, N. Weiskopf, B. Draganski

**Affiliations:** a LREN, Dept. of Clinical Neurosciences, CHUV, University of Lausanne, Lausanne Switzerland; b University Medical Centre, UMG, Dept. of Cognitive Neurology, Göttingen, Germany; c Wellcome Trust Centre for Neuroimaging, UCL Institute of Neurology, UCL, London, UK; d Max Planck Institute for Human Cognitive and Brain Sciences, Leipzig, Germany

**Keywords:** Quantitative magnetic imaging, Magnetization transfer, R1, Voxel-based morphometry, Voxel-based quantification, Basal ganglia

## Abstract

Evidence from magnetic resonance imaging (MRI) studies shows that healthy aging is associated with profound changes in cortical and subcortical brain structures. The reliable delineation of cortex and basal ganglia using automated computational anatomy methods based on T1-weighted images remains challenging, which results in controversies in the literature. In this study we use quantitative MRI (qMRI) to gain an insight into the microstructural mechanisms underlying tissue ageing and look for potential interactions between ageing and brain tissue properties to assess their impact on automated tissue classification. To this end we acquired maps of longitudinal relaxation rate R1, effective transverse relaxation rate R2* and magnetization transfer – MT, from healthy subjects (n = 96, aged 21–88 years) using a well-established multi-parameter mapping qMRI protocol. Within the framework of voxel-based quantification we find higher grey matter volume in basal ganglia, cerebellar dentate and prefrontal cortex when tissue classification is based on MT maps compared with T1 maps. These discrepancies between grey matter volume estimates can be attributed to R2* - a surrogate marker of iron concentration, and further modulation by an interaction between R2* and age, both in cortical and subcortical areas. We interpret our findings as direct evidence for the impact of ageing-related brain tissue property changes on automated tissue classification of brain structures using SPM12. Computational anatomy studies of ageing and neurodegeneration should acknowledge these effects, particularly when inferring about underlying pathophysiology from regional cortex and basal ganglia volume changes.

## Highlights

• We acquire quantitative bias free whole-brain maps of MT, R1 and R2* parameters.
• Grey matter volume estimation based on MT maps is higher than the R1-based in specific cortical and subcortical areas.
• Differences in grey matter volume estimations are correlated with iron concentration and further modulated by age.
• Iron decreases locally the grey-white matter contrast in R1 but not in MT maps.

## Introduction

The detailed in vivo assessment of brain anatomy and the particularly challenging delineation of subcortical structures are crucial for understanding the biological processes of brain development and healthy ageing. In this context, the accuracy of automated brain morphometry methods is also important for the comprehension of the pathophysiology of neurological and neuropsychiatric disorders with basal ganglia involvement, such as Parkinson's syndrome, Huntington's disease, dystonia, various tremor forms, Tourette's syndrome and schizophrenia (Utter and Basso, 2008). There is substantial controversy in the literature about the magnitude, directionality and neurobiological interpretation of basal ganglia structural changes in both healthy ageing and in diseased brain (Koolschijn et al., 2009, Mars et al., 2011, Zoons et al., 2012). The majority of these studies assessed basal ganglia anatomy using T1-weighted (T1w) imaging data, which turned out to be methodologically challenging (Wonderlick et al., 2009, Babalola et al., 2009). The difficulties in assuring accurate and robust computer-based delineation of the basal ganglia are mainly due to the presence of a high amount of iron (Hallgren and Sourander, 1958), which results in poor and variable contrast on T1w magnetic resonance images (Patenaude et al., 2011, Callaghan et al., 2014).

Previous research has demonstrated that MT saturation maps provide better contrast in subcortical structures compared to T1w images, resulting in improved automated tissue classification of thalamus and basal ganglia (Helms et al., 2009). The origin of this discrepancy remains unclear and could arise from differential contributions of tissue microstructural properties to image contrast or from the presence of spatial bias in the T1w data. In this study we explore the microstructural origin of these contrast differences using bias corrected quantitative MRI (qMRI) data. We examine the impact of each microstuctural contribution to the grey matter (GM) volume estimates extracted from automated tissue classification of cortex and subcortical structures.

Iron is known to be particularly abundant in the basal ganglia, where it accumulates during ageing (Ogg and Steen, 1998a). MRI is a valuable tool to estimate iron levels in vivo (Aquino et al., 2009, Langkammer et al., 2010) and to assess its distribution in the brain in normal ageing (Haacke et al., 2010) and in neurodegenerative diseases (Dumas et al., 2012). The multi-parameter (MPM) qMRI protocol used in our study provides estimates of the MRI parameters MT, R1 (= 1/T1), R2* (= 1/T2*) and proton density (PD) (Weiskopf et al., 2013). It has recently been used to introduce a General Linear Model of R1 in vivo using MT and R2* as surrogate markers of macromolecular and iron concentration (Callaghan et al., 2014b). Macromolecular and iron contents were shown to be the primary contributors to spatial variations of R1 across the brain. In this study, we therefore hypothesize that changes in iron concentration are a possible dominant factor behind the apparent change in GM volume during ageing. An additional goal of our study is to test for potential interactions between the differential estimation of GM volume and iron accumulation in the brain at different ages.

We set out to test if age-related changes in tissue properties introduce bias into automated delineation of basal ganglia structures using computational anatomy methods. To do this, we performed voxel-based morphometry (VBM) (Ashburner and Friston, 2000) and voxel-based quantification (VBQ) (Draganski et al., 2011) in the framework of statistical parametric mapping (SPM) (Wellcome Trust Centre for Neuroimaging, London, UK; http://www.fil.ion.ucl.ac.uk/spm). We used the established MPM quantitative imaging protocol providing whole-brain high resolution maps of R1, R2* and MT (Weiskopf et al., 2013), corrected for transmit inhomogeneity (Lutti et al., 2012), diffeomorphic registration (Ashburner, 2007), and VBQ analysis (Draganski et al., 2011). We correlated the differences in GM volume estimates extracted from the R1 and MT maps with age-dependent changes in R2*, a surrogate marker of iron content (Helms et al., 2008a, Langkammer et al., 2010).

## Methods

### Data acquisition

96 healthy adults (40 male, age range 27–74 years, mean 55 ± 15; 56 female, age range 21–88 years, mean 57 ± 19) were examined on a 3 T whole-body MRI system (Magnetom TIM Trio, Siemens Medical Systems, Germany), using a standard 32-channel RF receive head coil and body coil for transmission. Part of the data we used were previously acquired for a study on the effects of healthy ageing and tissue property changes (Chowdhury et al., 2012, Callaghan et al., 2014). Study participants showed neither macroscopic brain abnormalities, such as atrophy, nor signs of overt vascular pathology, such as micro-bleeds or white matter hyperintensities; participants with significant atrophy or white matter hyperintensities of Grade 2 or more by the Scheltens' rating scale (Scheltens et al., 1993) were excluded from the study. Informed written consent for anonymized data use in multiple studies was obtained according to a local Ethics committee approved protocol.

The quantitative MPM acquisitions consisted of three multi-echo 3D fast low angle shot (FLASH) with PD (TR/*α* = 23.7 ms/6^0^), T1 (TR/*α* = 18.7 ms/20^0^), and MT (TR/*α* = 23.7 ms/6^0^)-weighted contrast (Helms et al., 2008b). The MT-weighted contrast was obtained using an off-resonance Gaussian MT saturation pulse of 4 ms duration with a 220^0^ nominal flip angle, 2 kHz frequency offset. The field of view was (240 mm, 256 mm and 176 mm) along the (A-P, H-F and L-R) directions and the image resolution was 1 mm^3^ isotropic. Parallel imaging along the phase encoding (acceleration factor 2, GRAPPA image reconstruction) and Partial Fourier 6/8 in the partition direction were used to speed up data acquisition. The total acquisition time was 23 minutes.

Quantitative MRI maps were calculated from the acquired data using SPM12 running under Matlab 7.11 (Mathworks, Sherborn, MA, USA). In brief, regression of the log-signal from the eight PD-weighted echoes was used to calculate a map of R_2_*. The signals of six equidistant bipolar gradient echoes (at 2.2–14.7 ms echo time) were averaged to increase the signal-to-noise ratio (SNR), (Helms and Dechent, 2009) before calculation of the MT, R1 and effective proton density (PD*) maps as described in (Weiskopf et al., 2013). Note that here we refer to proton density (i.e. water concentration) as PD* to reflect the contribution of R_2_* to this parameter due to the echo averaging. To correct for the effects of radio frequency (RF) transmit inhomogeneities on the qMRI data, maps of the transmit field, B1 +, were obtained from a 3D echo-planar imaging (EPI) spin-echo (SE)/stimulated echo (STE) method described in (Lutti et al., 2010, Lutti et al., 2012).

### Data processing

#### Grey matter volume estimates – voxel-based morphometry

For VBM analysis, the MT saturation and R1 maps were processed independently in SPM12 with the same default settings and classified into different tissue classes: GM, white matter (WM), cerebral-spinal fluid (CSF) and non-brain tissue using the segmentation approach (Ashburner and Friston, 2005). Aiming at optimal anatomical precision we applied the diffeomorphic registration algorithm DARTEL (Ashburner, 2007) with default settings on the grey and white matter tissue maps derived from the MT and R1 images. The warped GM probability maps were scaled by the Jacobian determinants of the deformation fields to account for local compression and expansion due to linear and non-linear transformation (Ashburner and Friston, 2000), resulting in GM volume maps. The GM volume maps were then smoothed by convolution with an isotropic Gaussian kernel of 6 mm full-width-at-half-maximum (FWHM). Following the logic of the matched filter theorem (Jones et al., 2005, Salmond et al., 2002), we decided for a smoothing kernel width, which matches the spatial scale of the expected ageing-related volume differences. The GM volume maps were then smoothed by convolution with an isotropic Gaussian kernel of 6 mm full-width-at-half-maximum (FWHM).

#### Iron measurements – voxel-based quantification

For VBQ analysis the MT, R1 and R2* parameter maps were warped to standard MNI space using the subject-specific diffeomorphic estimates from the DARTEL procedure of the previous step, without scaling by the Jacobian determinants. A combined probability weighting and Gaussian smoothing procedure (Draganski et al., 2011) was used with a 6 mm FWHM isotropic smoothing kernel. This method results in spatial registration of parameter maps to MNI space, while optimally preserving the quantitative parameter values within each tissue class by reducing any effects of residual registration problems and partial volume.

### Statistical analysis

For investigation of regional differences between GM volume derived from the MT and R1 maps, we used a paired t-test with a whole-brain search volume defined by the automated anatomical labeling (AAL), human brain atlas (Tzourio-Mazoyer et al., 2002), the SUIT atlas of cerebellum and brainstem (Diedrichsen, 2006), and the basal ganglia human area template (BGHAT) (Prodoehl et al., 2008). Regional differences between MT and R1 GM segments were examined by creating voxel-wise statistical parametric maps (SPMs) for the whole extent of the search volume using the General Linear Model (GLM) and Random Field Theory (Friston et al., 1994). Significance level was set at *p* < 0.05 with family-wise error (FWE) correction for multiple comparisons.

### Multiple regression model

To test the hypothesis that differences in estimated GM volumes are a function of the underlying brain tissue properties, we carried out a linear regression between GM maps, iron content and age. Given the number of investigated study participants – 96, and their age range – 21–88 years old, we extended the model to include also potential non-linear ageing effects (Walhovd et al., 2011). The multiple regression was performed on a voxel-by-voxel basis beyond the statistical threshold *p_FWE_* < 0.05 in the paired t-test described above.

We first calculated the differences between GM volume estimated from and MT and the one estimated from R1 data. This was followed by a multiple regression analysis explaining GM volume differences by iron content, age, their interaction and the simultaneous contribution of age^2^. The correlation was modeled as:

(1) $$\mathrm{\Delta GM}=\beta_{1\;}\left[\mathrm{Fe}\right]+\beta_{2\;}\mathrm{age}+\beta_{3\;}\left[\mathrm{Fe}\right]X\;\mathrm{age}+\beta_{4\;}\mathrm{ag}e^{2}+\varepsilon$$

where ΔGM is the difference between the MT derived GM volume map and that computed from the R1 map; [Fe] – the iron content; β_n_ (with n = 1,2,3,4) – the coefficients weighting the contribution of iron concentration, age, the product of the two and the quadratic age effect on ΔGM; ε represents the residuals of the model.

We used a polynomial approach as recent studies on structural brain aging, that included nonlinearities supported the usage of polynomial models up to degree 3 (Walhovd et al., 2011). This procedure accounts for irregular intervals between measures (Wierenga et al., 2014).

Due to the linear relationship between R2* and iron content in iron-rich areas (Langkammer et al., 2010), Eq. (1) can be re-written as:

(2) $$\mathrm{\Delta GM}=\;\beta_{1\;}R_{2}^{*}+\beta_{2\;}\mathrm{age}+\beta_{3\;}R_{2\;}^{*}X\;\mathrm{age}+\beta_{4\;}\mathrm{ag}e^{2}+\varepsilon$$

All independent variables in Eq. (2) were mean centered.

The model was set to determine the β_n_ parameters and residuals at each voxel. To assess the quality of parameter estimation we computed T-values for each of them, testing against the null hypotheses that each β_n_ was equal to zero. The statistical significance level was set at *p_FWE_* < 0.05. The estimated regressors for differences in GM volume derived from MT and R1 maps were also tested for potential gender effects.

Considering the fact that the R2* parameter is related not only to iron content, but may also be influenced by the water content in brain tissue (Haacke et al., 2005), we computed the correlation coefficient between the R2* and the PD* maps on all voxels beyond the statistical threshold *p_FWE_* < 0.05 in the paired t-test described above. To further evaluate the relationship with the water content, we also studied the correlation between β_2_ and β_3_, respectively, with PD* values. All coefficients were computed using Pearson’s correlation within voxels exceeding the statistical threshold of *p_FWE_* < 0.05 in the paired t-test described for the GM volume estimates.

## Results

### Grey matter volume

On visual inspection substantia nigra, rostral pallidum, caudal putamen and thalamus showed higher contrast against the surrounding tissue on the MT maps compared to the R1 maps (see supplementary material Fig. 1). The very same regions showed higher GM estimates after automated tissue classification (see supplementary material Fig. 2).

**Fig. 1:**
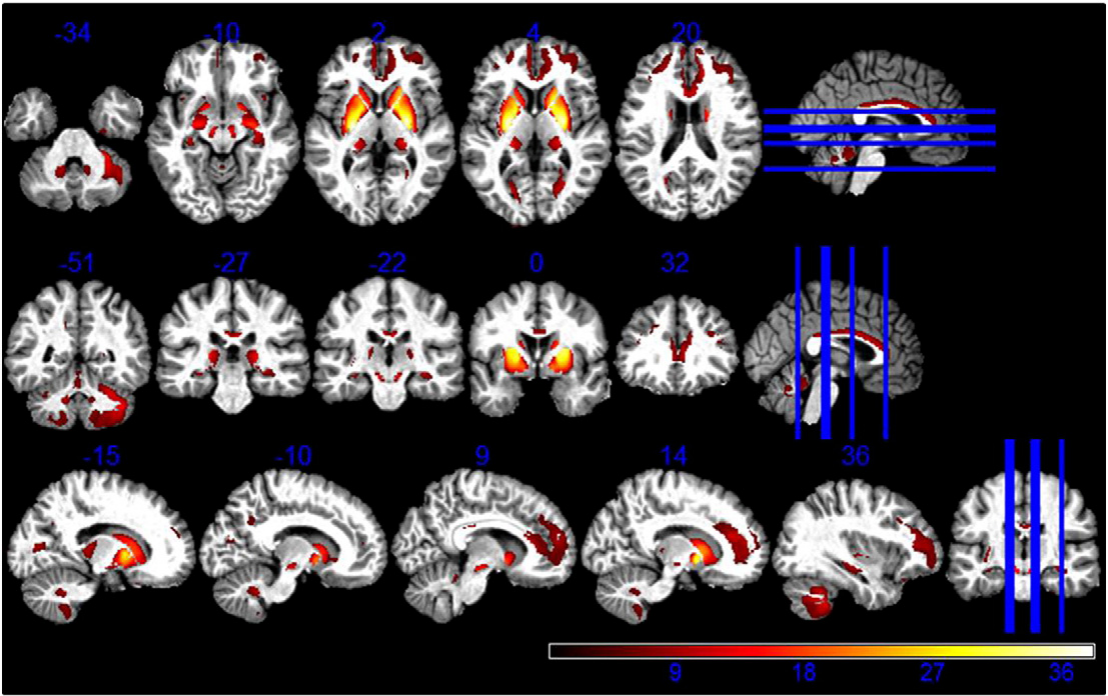
Higher GM volume estimation from MT maps compared to R1 maps. Statistical map of a paired t-test at statistical threshold of *p_FWE_ < 0.05* displayed on MT image in standard MNI space.

**Fig. 2:**
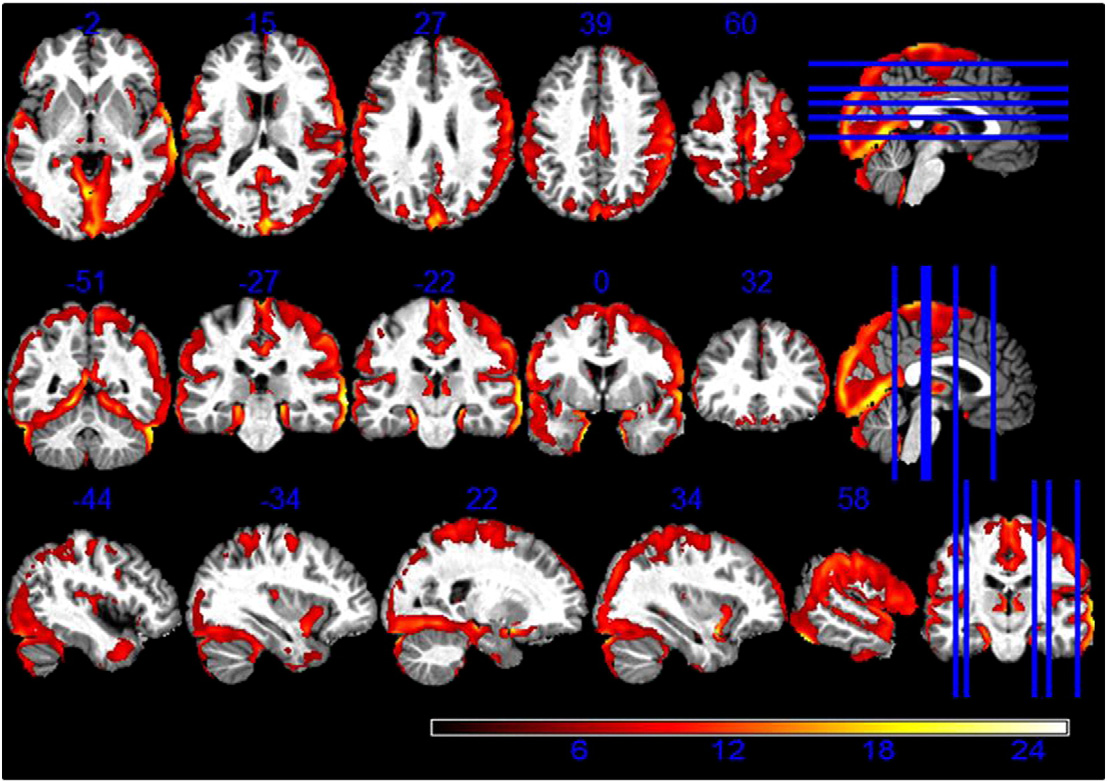
Higher GM volume estimation from R1 maps compared to MT maps. Statistical map of a paired t-test at statistical threshold of *p_FWE_ < 0.05* displayed on MT image in standard MNI space.

There was a significantly higher GM volume derived from MT maps in pallidum, putamen, lateral geniculate body of thalamus, substantia nigra, cerebellar dentate, cingulate and prefrontal cortex compared to estimates derived from R1 maps, as reported in Table 1 (see Fig. 1). Regions close to the boundary between GM and CSF in the sensory-motor cortex showed lower GM volumes estimated from the MT saturation maps compared with the R1 maps (see Fig. 2).

**Table 1:** Comparison between R1- and MT-based GM volume maps. Coordinates in MNI standard space. SNc = substantia nigra pars compacta; GP = globus pallidus.

Analysis	Region	Left hemisphere coordinates (mm)			T-value	Right hemisphere coordinates (mm)			T-value
		x	y	z		x	y	z	
	Putamen	− 24	− 13	1	38.15	25	− 5	9	35.82
	Caudate	− 18	15	6	28.77	18	20	1	23.7
	GP	− 21	− 6	6	34.55	23	− 4	5	33.74
	SNc	− 6	− 16	− 15	17.55	6	− 16	− 15	16.28
MT > R1	Thalamus	− 23	− 27	9	15.28	24	− 27	0	13.27
	Dentate	− 12	− 51	− 35	8.00	12	− 51	− 35	7.00
	Prefrontal cortex	− 21	44	19	9.16	33	44	10	9.65
	Cingulate	− 15	36	4	6.21	17	41	18	12.37
R1 > MT	Sensory-motor cortex	34	− 7	61	15.21	45	− 22	64	19.57

### Correlation with R2*

**Fig. 3:**
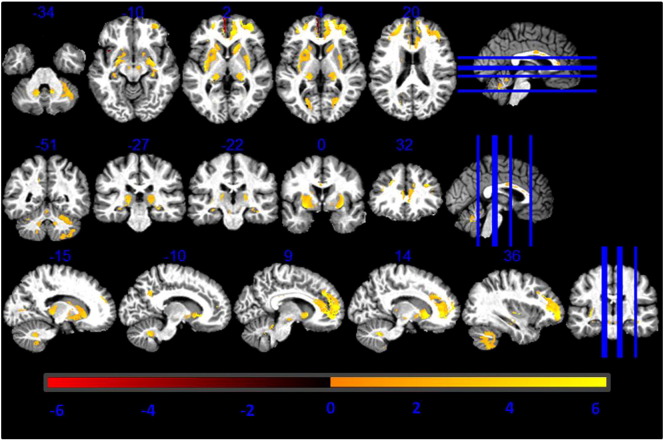
T-values of voxel-based regressors correlating higher GM volume estimation from MT maps compared to R1 maps with R2* values. Statistical map of a t-test at statistical threshold of *p_FWE_ < 0.05* displayed on MT image in standard MNI space.

**Fig. 4:**
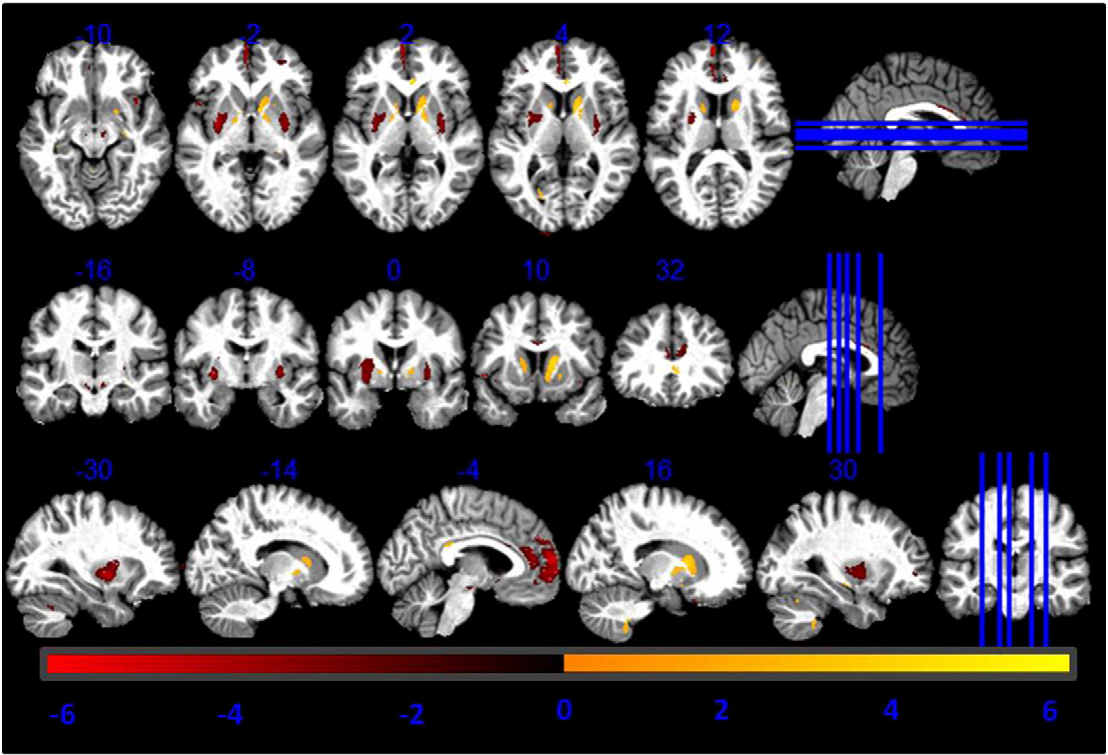
T-values of voxel-based regressors correlating higher GM volume estimation from MT maps compared to R1 maps with age. Statistical map of a t-test at statistical threshold of *p_FWE_ < 0.05* displayed on MT image in standard MNI space.

**Fig. 5:**
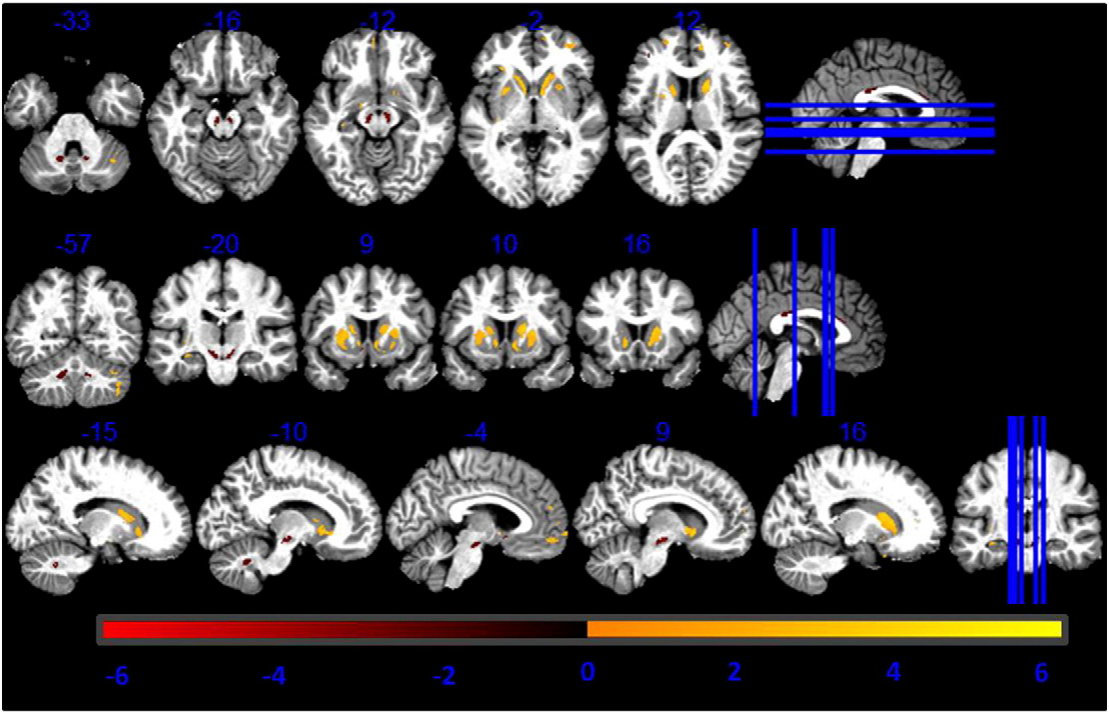
T-values of voxel-based regressors correlating higher GM volume estimation from MT maps compared to R1 maps with the interaction between R2* values and age. Statistical map of a t-test at statistical threshold of *p_FWE_ < 0.05* displayed on MT image in standard MNI space.

The GM volume differences between MT and R1 based GM volume estimations correlated positively with R2* maps in caudate, ventral part of putamen and pallidum, substantia nigra, lateral geniculate body of thalamus, cingulate, prefrontal cortex and dentate as reported in Table 2 (Fig. 3). The body of the caudate correlated positively with R2*, age and with the combined contribution of the two (see Fig. 3, Fig. 4, Fig. 5). Within our cohort the computed mean Pearson correlation coefficient between R2* and PD* was − 0.65 (p < 0.05).

**Table 2:** T-values of the regressors correlating GM differences between R1- and MT-based GM volume maps with R2* values. Coordinates in MNI standard space.

Region	Left hemisphere coordinates (mm)			T-value	Right hemisphere coordinates (mm)			T-value
	x	y	z		x	y	z	
Putamen	− 24	− 13	1	6.51	25	− 5	9	6.54
Caudate	− 18	15	6	4.75	18	20	1	6.1
GP	− 21	− 6	6	4.39	23	− 4	5	4.19
SNc	− 6	− 16	− 15	3.09	6	− 16	− 15	3.45
Thalamus	− 23	− 27	9	4.0	24	− 27	0	3.0
Dentate	− 12	− 51	− 35	4.65	12	− 51	− 35	6.00
Prefrontal cortex	− 21	44	19	7.24	33	44	10	6.29
Cingulate	− 15	36	4	4.55	17	41	18	3.55

### Linear correlation with age

The linear regressors determined to link GM volume differences with age showed a negative correlation between these two in the dorsal part of the putamen and age as reported in Table 3 (see Fig. 4). There was no significant interaction between the regressor and the gender of study participants. The mean Pearson coefficient for the correlation between the age regressor and PD* was equal to 0.01 (p < 0.05).

**Table 3:** T-values of regressors correlating GM differences between R1- and MT-based GM volume maps with age. Coordinates in MNI standard space. SNc = substantia nigra pars compacta; GP = globus pallidus.

Region	Left hemisphere coordinates (mm)			T-value	Right hemisphere coordinates (mm)			T-value
	x	y	z		x	y	z	
Putamen	− 30	− 5	− 2	− 3.0	32	− 8	2	− 3.1
Caudate	− 15	11	12	3.2	11	8	2	4.56
GP	− 17	− 5	2	− 2.69	--	--	--	--
SNc	− 6	− 17	− 14	− 3.13	8	− 20	− 14	− 2.94
Prefrontal cortex	− 5	59	11	− 3.7	12	32	26	− 2.7

To assess whether there was a differential effect of ageing on GM volume estimates from MT and R1 maps, we applied the regression model – expressed by Eq. (2) – separately to GM volume maps derived from MT and R1 using as covariates the R2* values, subject age and a combination of the two.

**Fig. 6:**
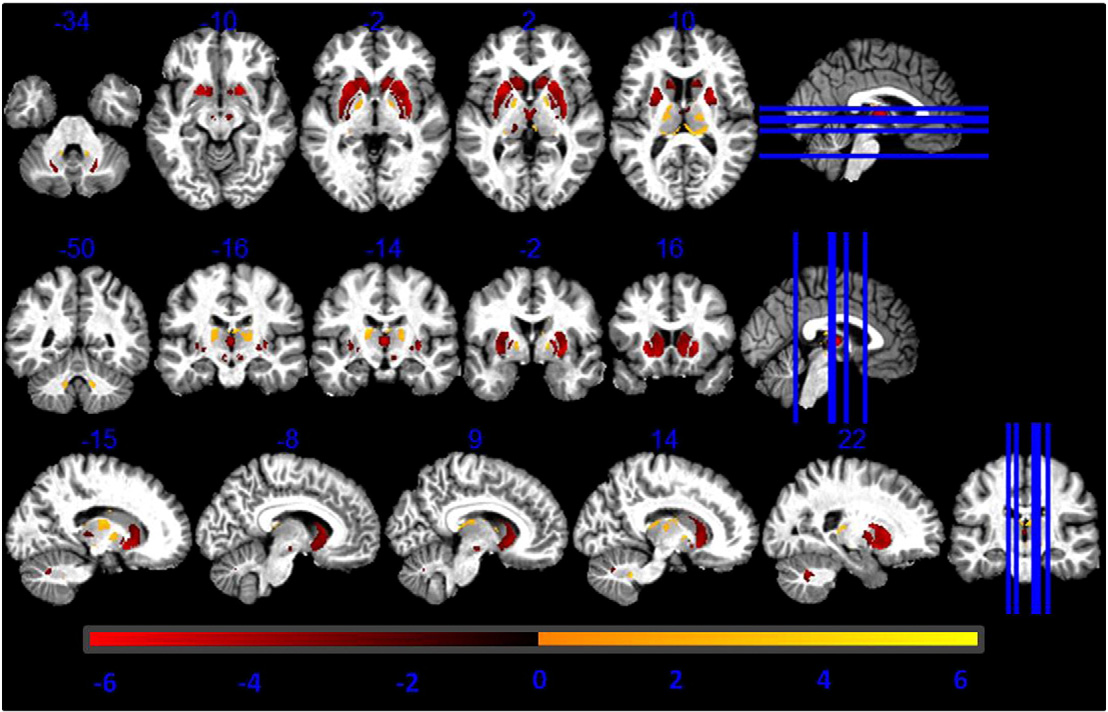
T-values of regressors correlating the GM volume estimated from MT maps with age. Statistical map of a t-test at statistical threshold of *p_FWE_ < 0.05* displayed on MT image in standard MNI space.

**Fig. 7:**
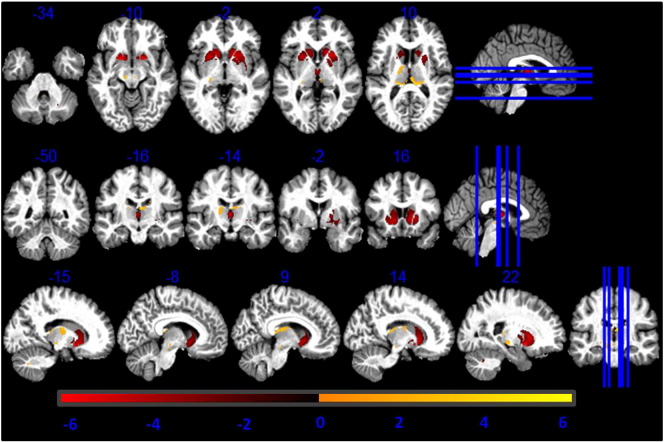
T-values of regressors correlating the GM volume estimated from R1 maps with age. Statistical map of a t-test at statistical threshold of *p_FWE_ < 0.05* displayed on MT image in standard MNI space.

The statistically significant results of this linear model were a negative correlation between age and MT derived GM volume maps in the whole putamen and the lateral part of the pallidum (see Fig. 6). The same linear model applied to R1 derived GM volume maps showed a negative correlation with age only in the anterior part of putamen and pallidum (see Fig. 7). We observed a significant negative correlation between age and regions where a greater GM volume was estimated with R1 than MT data.

**Fig. 8:**
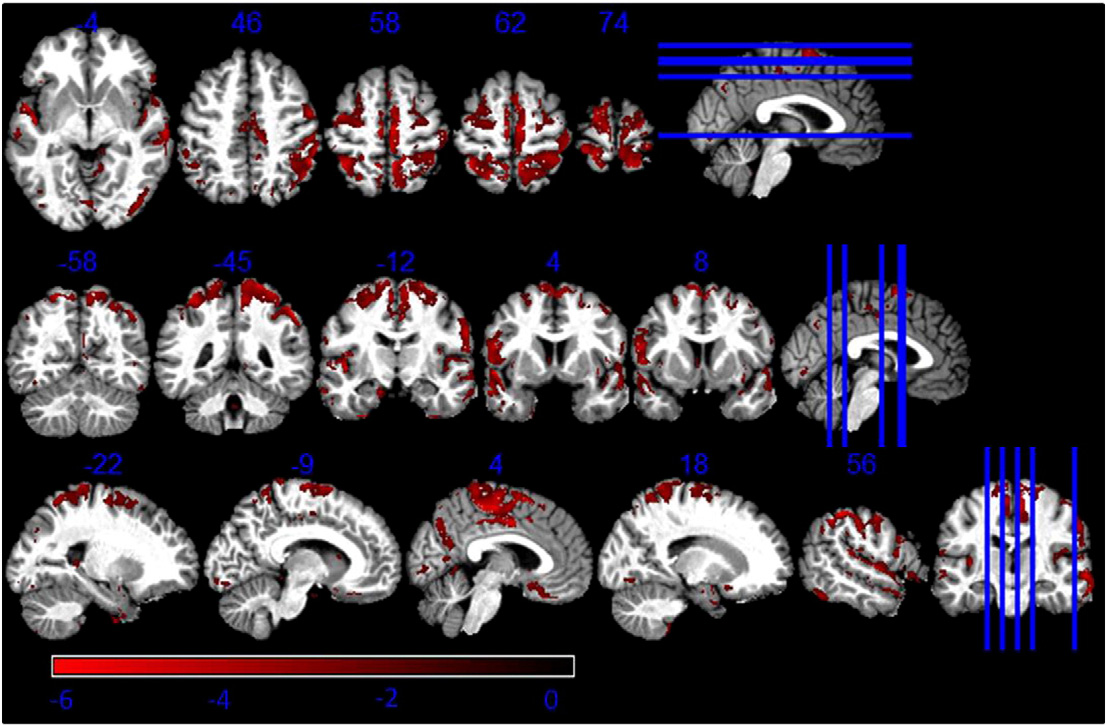
T-values of regressors correlating lower GM volume estimation from MT maps compared to R1 maps with age. Statistical map of a t-test at statistical threshold of *p_FWE_ < 0.05* displayed on MT image in standard MNI space.

We also detected a significant negative correlation between age and regions where GM volume estimated from R1 maps was greater than volume estimated from MT maps (see Fig. 8).

### Correlation with age-dependent R2* changes

The linear regressors weighting the simultaneous contribution of age and R2* showed that GM volume differences in the head of caudate and in the ventral part of putamen were positively correlated with age and R2*, as shown by Fig. 5. The substantia nigra and the cerebellar dentate were negatively correlated with these two parameters, as reported in Table 4. There was no significant interaction between the regressor and the gender of study participants. The mean Pearson coefficient for the correlation between the age regressor and PD* was not significant (p = 0.07).

**Table 4:** T-values of regressors correlating GM differences between R1- and MT-based GM volume maps with the interaction between R2* values and age. Coordinates in MNI standard space. SNc = substantia nigra pars compacta; GP = globus pallidus.

Region	Left hemisphere coordinates (mm)			T-value	Right hemisphere coordinates (mm)			T-value
	x	y	z		x	y	z	
Putamen	− 24	− 13	1	5.9	25	− 5	9	5.4
Caudate	− 18	15	6	4.85	18	20	1	4
GP	− 21	− 6	6	4.9	23	− 4	5	3.79
SNc	− 6	− 16	− 15	− 3.42	6	− 16	− 15	− 4.18
Dentate	− 12	− 51	− 35	− 3.5	12	− 51	− 35	− 3

### Non-linear correlation with age

There was no significant correlation between the quadratic age term and the GM volume estimated from MT and R1 maps. We found a positive correlation between the quadratic term of the age and GM volume differences between estimates from MT and R1 maps in the lateral geniculate of the thalamus and in the head of the caudate, as reported in Table 5.

**Table 5:** T-values of regressors correlating GM differences between R1- and MT-based GM volume maps with the quadratic contribution of age. Coordinates in MNI standard space.

Region	Left hemisphere coordinates (mm)			T-value	Right hemisphere coordinates (mm)			T-value
	x	y	z		x	y	z	
Caudate	− 15	− 2	17	4.3	17	17	14	3.8
Lateral geniculate of the thalamus	− 20	− 29	5	2.9	18	− 26	5	4.17
Cingulate	− 2	35	11	− 3.2	--	--	--	--

## Discussion

Our study using quantitative MRI multi-parameter mapping protocol demonstrates a strong link between brain tissue properties and the accuracy of automated tissue classification. The increase in probability of GM classification and hence estimated volume in basal ganglia, thalamic pulvinar, prefrontal cortex and cerebellum when comparing estimates from MT saturation and R1 maps is explained by differential effects of age, iron content and the interaction between these two quantities. Our findings showing both age-dependent and age-independent effects of brain iron on the estimation of GM volume in cortex and subcortical structures confirm the need to acknowledge their impact in morphometry studies of ageing and neurodegeneration.

Firstly, we confirm previous results demonstrating increased volume in pallidum, putamen, thalamic pulvinar and substantia nigra when using automated tissue classification of MT saturation maps compared with R1 derived GM estimates. Our analysis extends these findings to show increased volume estimation in the cerebellar dentate, anterior cingulate and prefrontal cortex in MT derived GM volume maps. A possible explanation for these additional findings, not reported previously, is increased statistical power due to a larger number of observations (n = 96 vs n = 49) and improvements in automated tissue classification algorithms implemented in SPM12 compared to SPM5 (Ashburner and Friston, 2005).

The novel aspect of our study is the investigation of the impact of brain iron on the accuracy of GM classification using a voxel-based multiple regression model. We test age-dependent and age-independent effects of iron content on the differences in GM estimates from MT saturation and R1 maps. According to our *a priori* hypothesis we find age-independent effects with higher MT derived GM volume estimates in the iron rich structures, e.g. basal ganglia and thalamus compared with R1 derived GM. The positive correlation between GM volume estimation differences and R2* maps in basal ganglia, thalamus, cerebellar dentate, cingulate and prefrontal cortex can be interpreted as an effect of iron content on the R1 map, which is not present in the MT maps. The resulting loss in contrast between grey and white matter in the R1 maps induces a systematic bias in automated tissue classification, leading to an apparent loss in GM volume in ageing. On the other hand the MT saturation maps remain unaffected by the presence of paramagnetic ions (Helms et al., 2008b, Draganski et al., 2011, Callaghan et al., 2014), which from a histological perspective, are known to accumulate in subcortical structures, particularly in pallidum and substantia nigra, in the process of healthy aging (Ogg and Steen, 1998) and brain disease (Dexter et al., 1991, Schenck et al., 2006, Berg and Youdim, 2006).

Our findings in the prefrontal cortex, reporting GM differences that are positively correlated with R2* values, can be attributed to iron concentration variability within prefrontal areas, rather than to volume differences (Zhou et al., 2001). Perl’s iron stains of brain sections from a rhesus monkey (Bizzi et al., 1990) and humans (Morris et al., 1992) indicate a cortical depth gradient of iron concentration with deeper layers having the highest iron concentration. This observation coincides with the higher myelin content in deeper cortical layers (Duyn et al., 2007). We can only speculate about the regional specificity of our findings in prefrontal cortex given that this part of the brain is the last to complete the process of intracortical myelination with myelin that is qualitatively different from that in early myelinating cortical areas (Rajkowska and Goldman-Rakic, 1995).

Secondly, our results confirm the co-existence of age-dependent volume changes next to specific effects of iron content on GM volume estimates. The characteristic pattern of age-dependent GM differences extends predominantly to subcortical structures of the “motor” cortico-basal ganglia-cerebellar circuits (Accolla et al., 2014). This is in line with previous reports about trends for negative correlation between MT-based GM estimates and age in the dorsal caudate and in the dorso-lateral putamen (Draganski et al., 2011). As the contrast in MT maps is mainly driven by myelin content (Vavasour et al., 1998) and the GM estimation is based on image contrast, we can infer that decreased GM volume may be partly explained by a loss of myelin content. The negative correlation between GM volume estimation with age supports the idea that MT reduction reflects structural change (Gringel et al., 2009) due to cell loss (Tambasco et al., 2003). Despite the large number of study participants, we observed a significant interaction effect between the quadratic age term and the GM volume differences between MT and R1 maps based GM volume estimates. This might be explained by the fact that although our cohort included participants from 21 to 88 years, the span between 40 and 70 years was more sparsely sampled, which is likely to have made our analysis particularly sensitive to linear rather than higher order term effects (Ziegler et al., 2012). The non-linear age effects on GM volume in the caudate and lateral geniculate of the thalamus are consistent with longitudinal findings reporting greater rate of ageing related atrophy in these regions (Raz et al., 2005).

Further, the complex relationship between structural brain features shown by our voxel-based regression model is not only due to the main effects of R2* and age, but also to an interaction between the two. We show differential anatomical patterns corresponding to positive and negative correlations between GM differences and the joint contributions of R2* and age. Thus, we find positive correlations between GM differences, R2* and age in the head of caudate and in the ventral part of the putamen, and negative correlations in the substantia nigra and cerebellar dentate. Post mortem histological studies (Hallgren and Sourander, 1958) and MR studies are consistent with age-dependent increased iron content (Bartzokis et al., 2007, Péran et al., 2009). Many studies have attempted to evaluate whether increased iron levels, measured by R2* values, are a marker for age-related cell loss with associated increases in iron concentrations, or whether the iron contributes to the pathogenesis of disease and to normal aging, as others have suggested (Jellinger, 1999, Connor et al., 1992). Age- and iron- related effects on GM volume cannot be solely explained by an increased sensitivity of R1 contrast to iron concentration. A number of tissue changes, for example increased water content, can be associated with healthy aging (Bartzokis et al., 1994) and neurodegenerative disorders (Jernigan et al., 1991). Thus, the observed correlations between GM volume differences and the age-dependent R2* changes most probably reflect a combined effect of different brain tissue property changes. This is confirmed by the strong correlation between R2* and PD* local values.

We also report age-related relative GM volume decreases when comparing MT and R1 derived values. This finding shows age related GM volume differences predominantly located in early myelinated regions of the primary sensorimotor cortex. Given the abundance of highly myelinated fibers in primary sensorimotor areas we interpret such age-dependent cortical volume differences as a possible reflection of a differential sensitivity of the two modalities of measurement to myelin microstructural patterns (Sereno et al., 2012).

### Limitations and outlook

Despite the neurobiological and anatomical plausibility of our findings, there are certain methodological limitations of note. Most importantly, the subject’s head position in the MRI scanner clearly has an impact on measured R2* values (Bender and Klose, 2010), which has a potential effect on the correlation between measured R2* values and iron content. This is of particular importance for studies of patients with movement disorders, whose head position in the scanner may be systematically different from that of healthy subjects. Another area of potential limitation is the accuracy of the spatial tissue priors used for automated tissue classification. To avoid potential bias, in our study we used the very same priors for tissue classification of MT and R1 maps. However, we cannot exclude interaction effects between contrast differences and prior information about the location of different tissues in the brain. We cannot avoid the potential bias due to partial volume effects assuming region-specific co-existence of age-dependent atrophy and spatial registration errors. We minimized such potential sources of bias by using a 20% threshold on the GM tissue maps used to compute statistical analyses.

Our results are particularly relevant for studies focusing on basal ganglia regions. The patterns of increased GM volume estimation was assessed using conventional mass univariate statistical analysis (Friston et al., 2002). This is a powerful approach, since it allows unbiased whole-brain analysis of large datasets and reliably controls the statistical family-wise error. We explain the differences in GM volume estimation as a function of microstructural and age-related patterns, showing that the contrast in MT maps is not affected by the presence of iron. On the basis of these considerations we confirm the suitability of MT maps for robust automated tissue classification, which is of particular relevance for morphometric studies focused on the basal ganglia. Also, this property can be used advantageously in the clinical domain, for example, to correctly target areas for DBS electrode implantation or for stereotactic surgery.
