# Supplementary material for: Disentangling in vivo the effects of iron content and atrophy on the ageing human brain
Source: Neuroimage. 2014 Dec;103:280–9. doi: 10.1016/j.neuroimage.2014.09.044 (PMC4263529; doi:10.1016/j.neuroimage.2014.09.044)
Supplement: Supplementary file 1 — Supplementary figures [file mmc1.docx]

**Supplementary material**

**
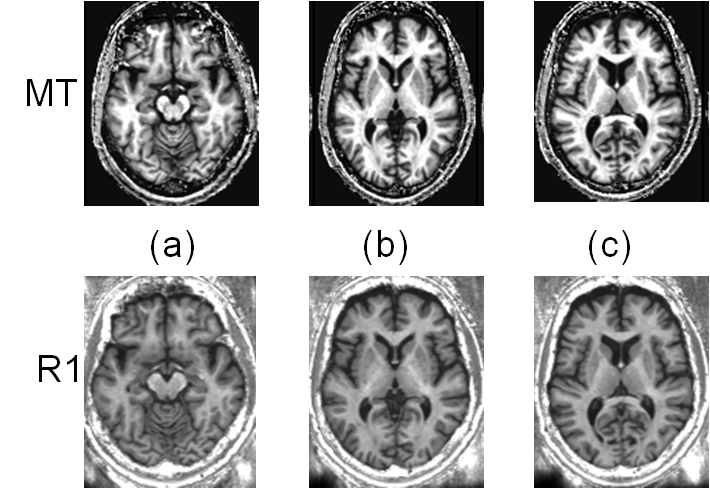
**

Figure 1 Example of single subject data: MT saturation maps (upper row) and R1 (R1=1/T1) maps (bottom row). Axial views at the level of: (a) substantia nigra, caudate nucleus, pallidum, putamen and thalamus (higher grey-white matter in the MT maps, (b) caudate, pallidum, putamen and thalamus, and (c) caudate, putamen and thalamus.


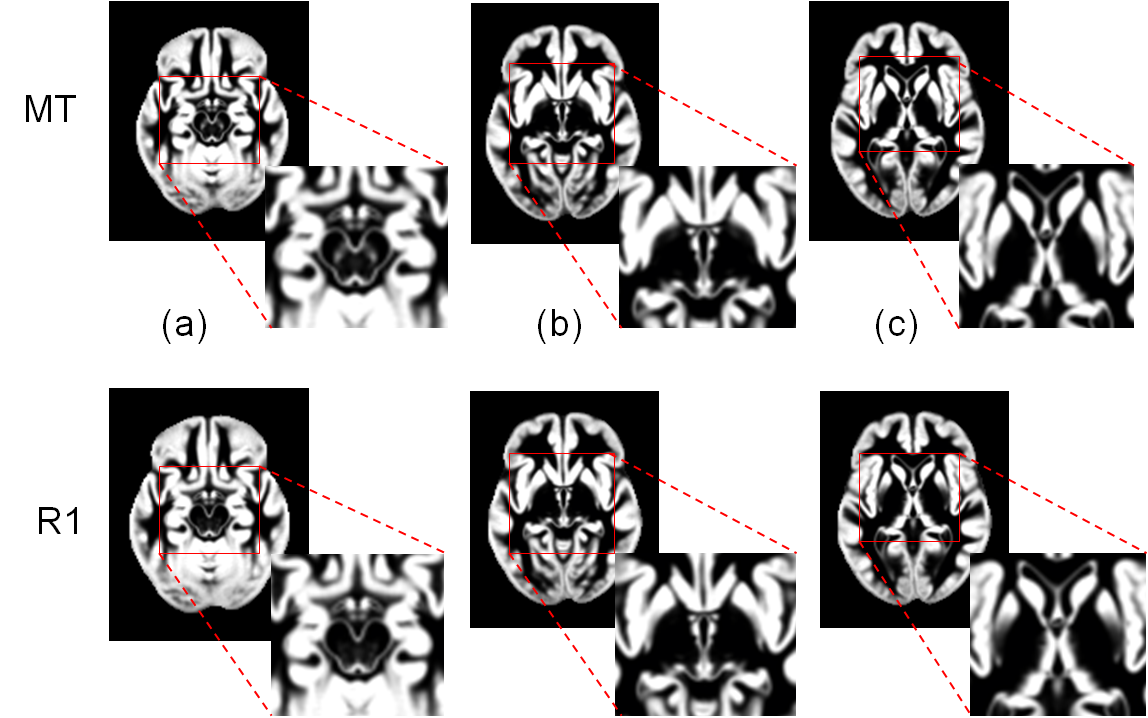


**Figure 2**: Average grey matter tissue maps, classified from MT- (upper row) and R1-maps (bottom row) in MNI standard space corresponding to axial views at the level of: (a) substantia nigra (b) pallidum, (c) putamen.
